# Characterization of a Novel Complete-Genome Sequence of a Galliform Chaphamaparvovirus from a Free-Range Laying Chicken Clinically Diagnosed with Spotty Liver Disease

**DOI:** 10.1128/mra.01017-22

**Published:** 2022-10-27

**Authors:** Subir Sarker

**Affiliations:** a Department of Microbiology, Anatomy, Physiology, and Pharmacology, School of Agriculture, Biomedicine, and Environment, La Trobe University, Melbourne, Victoria, Australia; DOE Joint Genome Institute

## Abstract

This study reports a novel complete genome of galliform chaphamaparvovirus 4, which was detected in the bile of a free-range laying chicken diagnosed with spotty liver disease. The genome was 4,367 bp in length, enclosed by two identical inverted terminal repeats. The detection of this novel chaphamaparvovirus represents a notable concern for the poultry industry in Australia.

## ANNOUNCEMENT

Chaphamaparvoviruses (ChPVs) are members of the family *Parvoviridae* and subfamiliy *Hamaparvovirinae* and are nonenveloped, icosahedral viruses with a linear single-stranded DNA genome of ~4.0 to 4.5 kb (1). They contain two major genes, namely, a nonstructural (NS) replicase gene and a capsid (VP) gene (2, 3). ChPVs are likely to be widespread in nature and have been detected in the feces of birds (4–8) and mammals (9), and a ChPV causes renal disease in laboratory mice (10). Recently, two novel ChPVs were detected in the livers of pheasants (Phasianus colchicus) (11) and in kidney tissue from a boobook owl (Ninox boobook) (12). Here, we report a novel complete genome of a ChPV, i.e., galliform ChPV 4 (GaChPV-4), that was detected in the bile of a free-range laying chicken that had been clinically diagnosed with spotty liver disease (SLD).

In 2021, a bile sample was collected from a chicken had been clinically diagnosed with SLD, from a free-range laying chicken farm in Seymour, Victoria, Australia. Dead chickens were necropsied by a registered veterinarian for routine diagnostic purposes. All other methods were performed in accordance with the standard guidelines and regulations for a physical containment level 2 (PC2) laboratory. The Animal Ethics Committee at La Trobe University was informed that findings from the diagnostic material were to be used in a publication, and a formal waiver of ethics approval was granted. Viral nucleic acids were extracted using a QIAamp viral RNA minikit (Qiagen, USA) without carrier RNA, which allowed the simultaneous extraction of viral DNA and RNA. The library was prepared using an Illumina DNA preparation kit, starting with 250 ng of DNA (6). The quality and quantity of the prepared library were assessed by the Australian Genome Research Facility (AGRF) (Melbourne, Australia), and the library was sequenced with the Illumina NovaSeq sequencing platform, generating 150-bp paired-end reads.

Sequencing data were analyzed with an established pipeline (13–16) using Geneious Prime (version 2022.1.1; Biomatters, New Zealand) and CLC Genomics Workbench (version 9.0.1). Briefly, 47.73 million raw reads were preprocessed to remove the Illumina adapters, ambiguous base calls, and poor-quality reads (trim using quality score limit of 0.05 and trim ambiguous nucleotides up to 15 using CLC Genomics Workbench), followed by mapping against the genomes of chickens (Gallus gallus) (GenBank accession number NC_006088.5) and Escherichia coli (GenBank accession number U00096) to remove nonviral DNA. A total of 45.5 million cleaned and unmapped reads were used as input data for the *de novo* default assembler in CLC Genomics Workbench (version 9.0.1). This resulted in the generation of a linear 4,367-bp contig, identified as a GaChPV-4 genome (based on similarity to the reference sequence of GaChPV-3 [GenBank accession number MW306779.1]), with average coverage of 178.1×. Annotation of the assembled genome was performed using Geneious Prime (version 2022.1.1). All software was used with default parameters except where stated.

**FIG 1:**
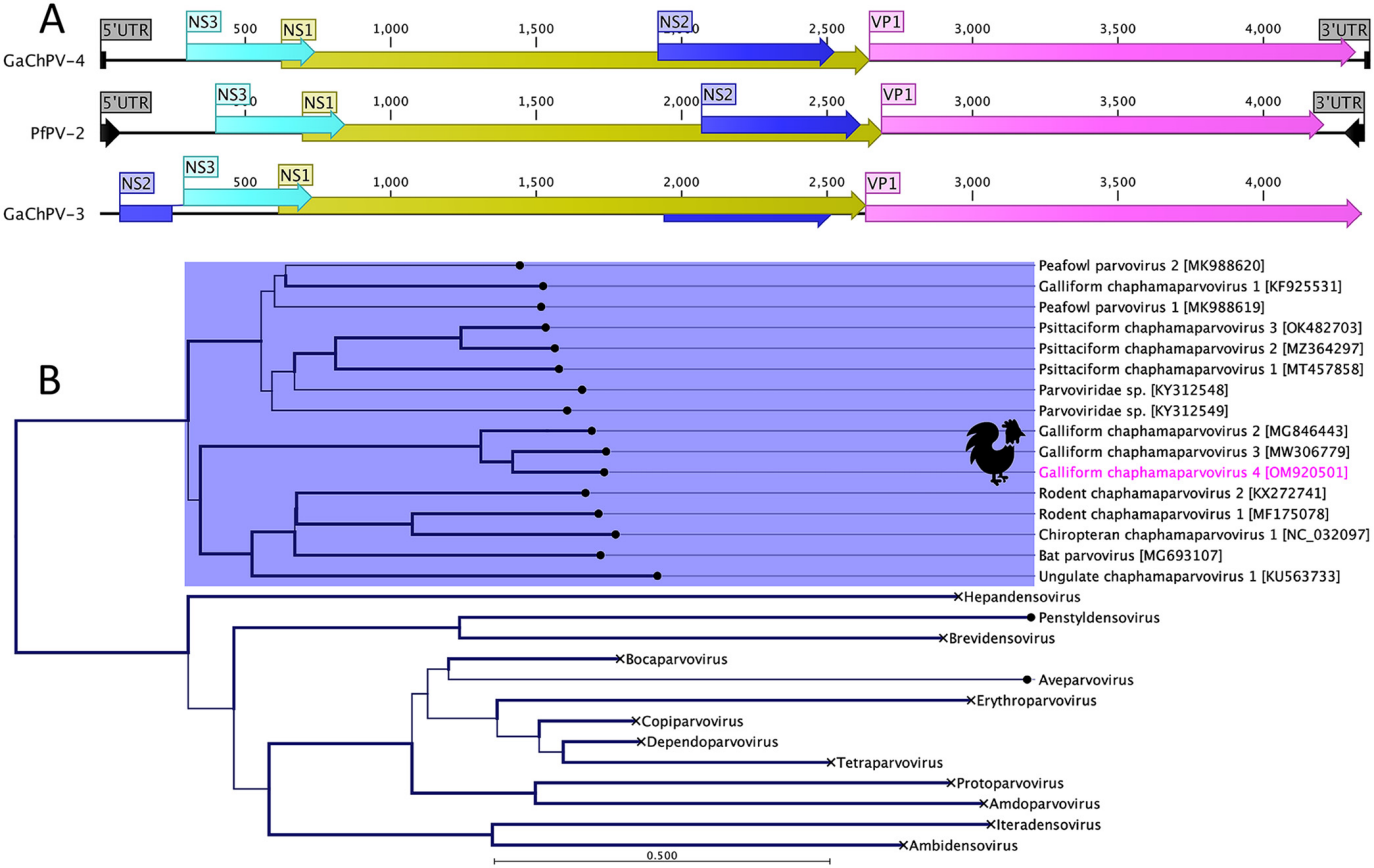
(A) Schematic illustration of selected GaChPVs. A schematic map of GaChPV-4 (GenBank accession number OM920501), compared to GaChPV-3 (GenBank accession number MW306779.1) and peafowl parvovirus 1 (PfPV-2) (GenBank accession number MK988620.1), was constructed using CLC Genomics Workbench (version 9.0.1; Qiagen, Hilden, Germany). The arrows symbolize ChPV genes and ORFs predicted to code for proteins, indicating their transcription direction. The genes and ORFs are color-coded as indicated. UTR, untranslated region. (B) Maximum likelihood phylogenetic tree using amino acid sequences of NS1 genes, showing the possible evolutionary relationships of the novel GaChPV-4 detected in this study to other selected parvoviruses. Amino acid alignment, followed by maximum likelihood tree construction, was performed using the option in CLC Genomics Workbench. The line weights indicate bootstrap values, and the labels at the branch tips refer to the original parvovirus species names, followed by the GenBank accession numbers in brackets. The clade corresponding to the ChPVs sequenced in this study has a blue background, and the GaChPV-4 sequenced in this study is shown in pink. All clades relevant to other genera are collapsed.

The genome is 4,367 bp long, with 21-nucleotide inverted terminal repeat sequences and with a G+C content of 39.9%. The GaChPV-4 contained four open reading frames (ORFs) (Fig. 1A), and comparative analysis of the predicted ORFs was conducted by using BLASTx and BLASTp with GenBank (17). The ORFs encoding NS1 and VP1 proteins share 77.63% and 70.02% amino acid identity, respectively, with the corresponding proteins of GaChPV-3 (GenBank accession number MW306779.1). Phylogenetically, GaChPV-4 shows obvious evolutionary relationships with other GaChPVs (Fig. 1B).

Like other parvoviruses, the complete NS1 gene of GaChPV-4 is 674 amino acids long and encodes a helicase, including the conserved ATP- or GTP-binding Walker A loop (GPxNTGKT/S [_322_**GP**S**NTGKS**_329_]), Walker B (xxxWEE [_360_WGK**WEE**_365_]), Walker B′ (KQxxEGxxxxxPxK [_377_**KQ**IA**EG**METHI**P**V**K**_390_]), and Walker C (PxxxTxN [_401_**P**IWI**T**T**N**_407_]) amino acid motifs. In addition, the NS1 protein contains two conserved replication initiator (endonuclease) motifs, i.e., xxHuHxxxx (IF_112_**H**V**H**_115_GLCK) and YxxK (_169_**Y**MC**K**_172_) (conserved amino acids are indicated in bold letters, and u indicates a hydrophobic residue).

This study reports evidence of a novel GaChPV-4 in a free-range laying chicken that was clinically diagnosed with SLD. Additional investigations will be required to better understand the host-pathogen dynamics, including routes of transmission, associated pathology, and disease prevalence.

### Data availability.

The complete GaChPV-4 genome sequence from the free-range laying chicken has been deposited in DDBJ/ENA/GenBank under the accession number OM920501. The version described in this paper is the first version, OM920501.1. The raw sequencing data from this study have been deposited in the NCBI Sequence Read Achieve (SRA) under the accession number SRR19134919 (BioProject accession number: PRJNA835504).
